# Achieving excellence with interdisciplinary approaches in complex orthodontic adult patients

**DOI:** 10.1038/s41415-024-7778-9

**Published:** 2024-09-13

**Authors:** Ute E. M. Schneider, Lorenz Moser

**Affiliations:** 41415209929001 University of Ferrara, Ferrara, Italy; University of Pennsylvania, Pennsylvania, USA; 41415209929002 University of Ferrara, Ferrara, Italy

## Abstract

Over the last decades the percentage of adult orthodontic patients has substantially increased. Undeniably, an important motif for seeking orthodontic care at an older age is smile improvement, but this is not all. Frequently, impaired dentofacial aesthetics are combined with several other issues: severe dentoskeletal malocclusions; multiple tooth loss due to caries or endodontic failure; dental agenesis or trauma; periodontal breakdown; or functional problems, such as temporomandibular disorders or obstructive sleep apnoea. Therefore, comprehensive adult treatment mostly requires close collaboration of a team of equally well-trained dental specialists to select and execute the most suitable treatment option for the individual patient from day one. With joint planning, intensive communication with the patient and the involved specialists, and continuous monitoring of the treatment process, true patient-centred care can be delivered. In order to serve the individual patient best, the treating orthodontist does not only need thorough speciality training and continuing education, but furthermore, should be well-equipped with sufficient knowledge of the other dental fields of expertise. In combination with digital technology as an important tool for enhancing communication and efficient exchange of information between all involved team members, excellent joint clinical skills will take comprehensive interdisciplinary treatment to the next level.

## Key points

- Treatment planning for the adult orthodontic patient should start on day one. Comprehensive, patient-centred orthodontic treatment should be delivered by an interdisciplinary team of equally well-trained and skilled specialists.
- Treating orthodontic adult patients to the highest standard of care requires more than a specialty degree in orthodontics but also sound knowledge of the related dental and medical disciplines to jointly plan, select and execute the most appropriate treatment approach for each patient in the most efficient way.
- Without close communication and continuous exchange of information between the team members interdisciplinary treatment is reduced to mere multidisciplinary care without coordination of the single procedures.
- Digital technology is a helpful tool to facilitate true patient-centred interdisciplinary treatment by continuous exchange of updated information and monitoring of the single therapeutic steps throughout the entire course of treatment.

## Introduction

While in the 1960s adults accounted for just 4% of patients, those numbers increased to 32% by 2022, and to date one-in-three orthodontic patients is an adult.^1^ The main motivation for undergoing orthodontic treatment is the desire to improve dental and smile aesthetics, missing teeth due caries, periodontal disease or trauma, and functional impairment are also typical clinical features for referring adults to the orthodontist. These patients present a multifaceted clinical picture requiring an interdisciplinary team to address the maximum number of highest priority problems, including the patient's chief complaint and to optimise the treatment results with the greatest benefit and the least risk by implementing evidence-based treatment approaches.^2^^,^^3^ Ideally, treatment planning and execution should start from the outset, with close collaboration of all involved specialists and the formulation of a well-structured problem list to ensure that all aspects have been evaluated in the diagnostic phase while also serving as a valuable reference tool during treatment.^4^^,^^5^^,^^6^

Pre-restorative orthodontics is directed at the most favourable distribution of teeth, with good root parallelism to optimise future restorative treatment, correction of occlusal plane for incisal guidance, improvement or correction of mucogingival and osseous defects, and at obtaining a more favourable crown-to-root ratio for long-term maintainability of periodontally compromised teeth. In the presence of severe skeletal discrepancies, a maxillofacial surgical approach might be necessary to establish normal inter-arch relationships for subsequent prosthodontic treatment.

Clinical examples will be used to illustrate the different indications for adult orthodontic-restorative treatment and the necessity for close collaboration of a well-orchestrated interdisciplinary team.

## Improvement of smile aesthetics

Excessive overjet, dental crowding or spacing; tooth discolouration or enamel dysplasia; a gummy smile; gingival recession with uneven gingival margins or open embrasures; and missing anterior teeth may negatively affect self-esteem, predisposing to psychosocial problems.^7^^,^^8^^,^^9^ If improvement of dental and smile aesthetics is the main motive for seeking treatment, visualisation of the result before implementing the definitive treatment plan may be considered ideal. The advent of digital smile design has enabled the clinician to develop a simulation of the envisioned treatment result which can be helpful both for communicating with the patient and the entire professional team.^10^^,^^11^^,^^12^^,^^13^^,^^14^ Moreover, sharing follow-up intraoral scans during treatment facilitates collaboration with the treating restorative dentist to orthodontically move the teeth in an ideal position for ‘minimally invasive‘ prosthodontic care.^15^^,^^16^^,^^17^^,^^18^ Delivering ultrathin feldspathic or disilicate ceramic laminate veneers may improve dental and smile aesthetics but also enhance the long-term success rate of these restorations.^19^^,^^20^

### Case example

**Fig. 1:**
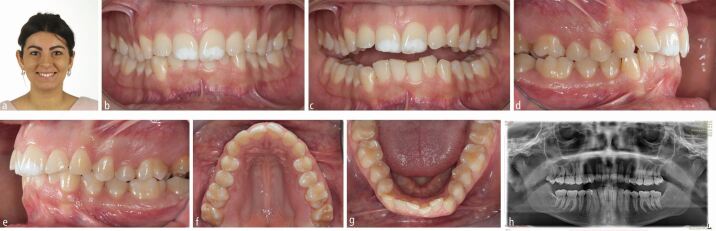
a, b, c, d, e, f, g, h) This 25-year-old female patient with constricted dental arches, maxillary and mandibular crowding, a steep lower curve of Spee, significant upper and lower anterior wear and hypo-mineralised upper central incisors requested an improvement of her smile aesthetics

A 25-year-old female patient presented with a mild skeletal II pattern; maxillary and mandibular anterior crowding; accentuated curves of Spee; enamel hypoplasia of the maxillary central incisors; severe dental wear of the upper and lower anterior teeth; and insufficient passive eruption of the maxillary incisors. Her chief complaint was discontent with her smile and nocturnal bruxism (Fig. 1).

**Fig. 2:**
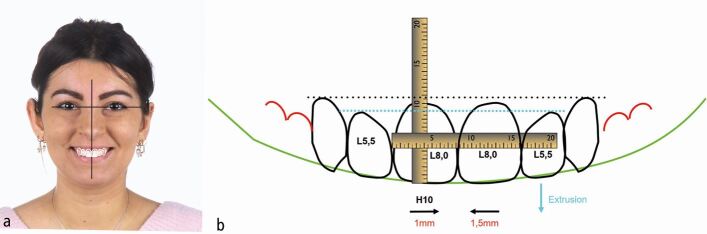
a, b) A digital smile design was performed to define tooth proportions and position

A joint interdisciplinary treatment plan, which included orthodontic levelling and alignment with fixed appliances; surgical crown lengthening of the maxillary incisors; six upper anterior feldspathic laminate veneers; and reconstruction of the worn lower incisor and canine cusps with direct composite restorations, was formulated. A digital smile design was presented to the patient for motivation and for explanation (Fig. 2).

**Fig. 3:**
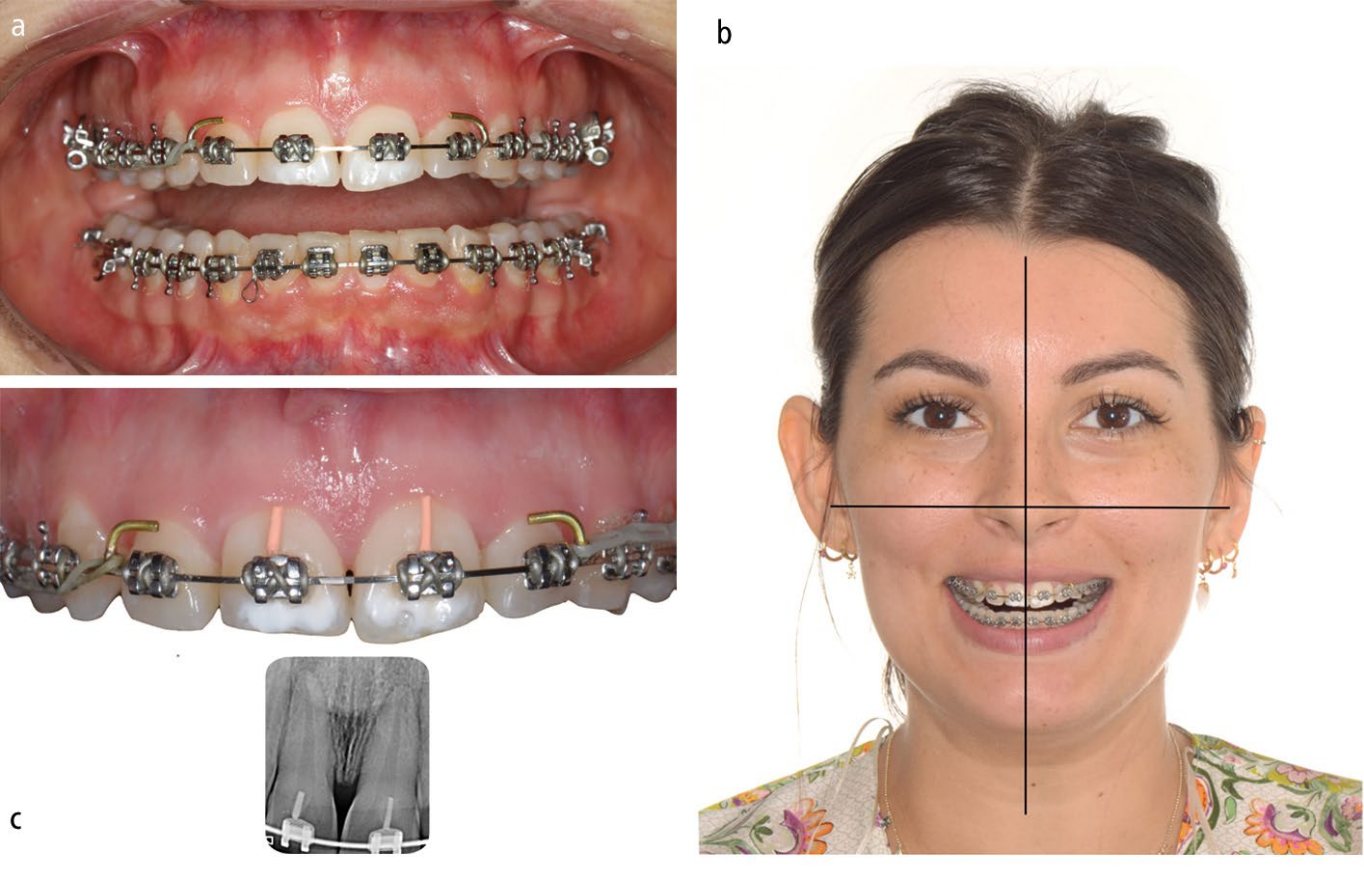
a, b, c) After complete levelling and aligning of the dentition, all aspects of macro-, mini- and micro-aesthetics and the necessary finishing procedures were discussed with the restorative dentist

**Fig. 4:**
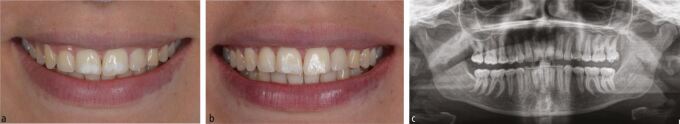
a, b, c) After clinical and radiographic assessment of the position of the cementoenamel junction, surgical crown lengthening was carried out for creating harmonious gingival contours and a consonant smile line once the appliances were removed

**Fig. 5:**
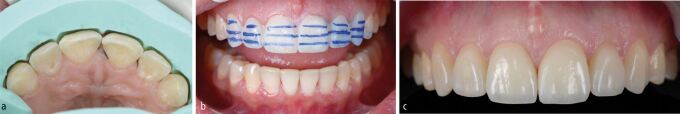
a, b, c) After orthodontics, only minimal preparation is necessary for the insertion of six ultrathin feldspathic ceramic veneers

Treatment started with bonding of a straight-wire 0.022-inch appliance (MBT prescription) for levelling and aligning of the dental arches. During the orthodontic finishing phase, close interaction with the restorative dentist and the periodontist to define the ideal final tooth position and manage the gingival margins by surgical crown lengthening was necessary to minimise enamel preparation and to reduce the biological cost of treatment (Fig. 3, Fig. 4). Following minimal enamel preparation, six ultrathin feldspathic ceramic veneers were placed (Fig. 5).

**Fig. 6:**
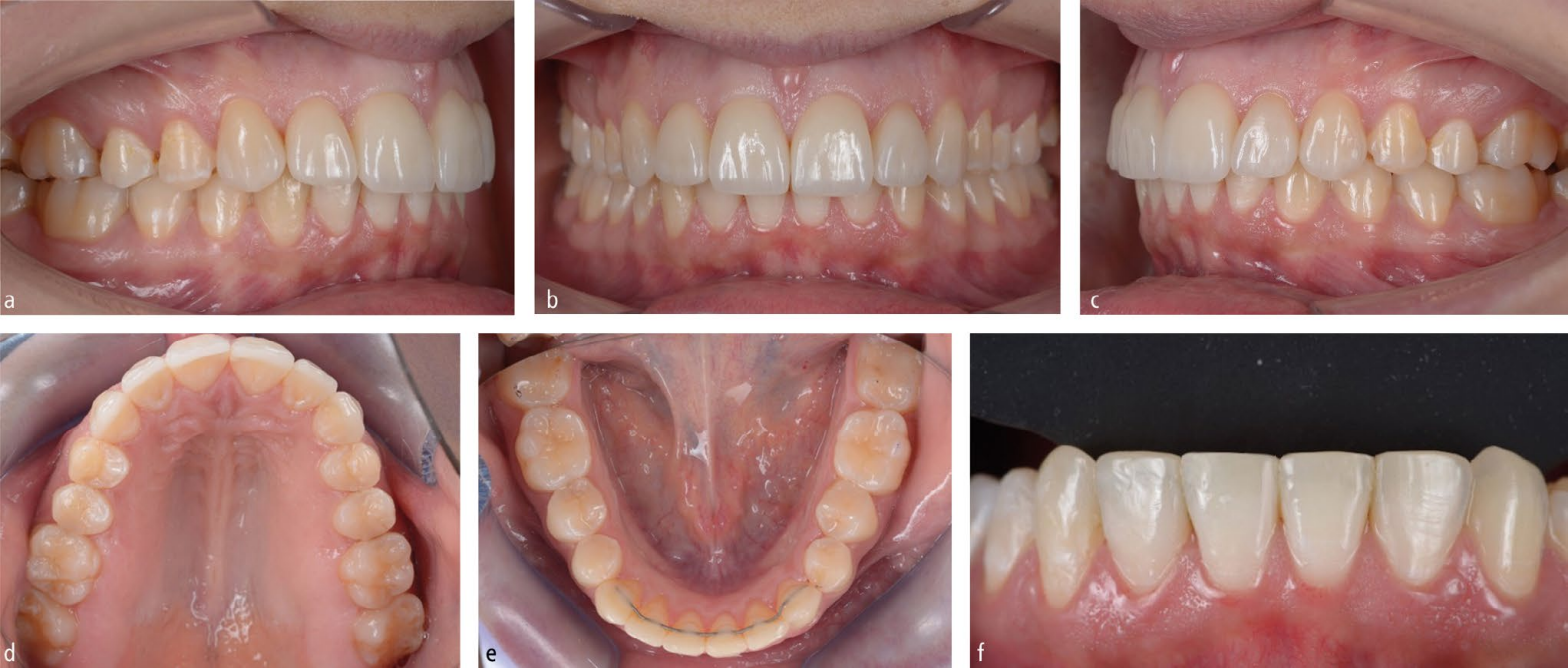
a, b, c, d, e, f) Together with the delivery of six anterior veneers, it was mandatory to also restore the worn lower incisal edges and canine cusps with composite material to ensure maintainability of the vertical dimension

**Fig. 7:**
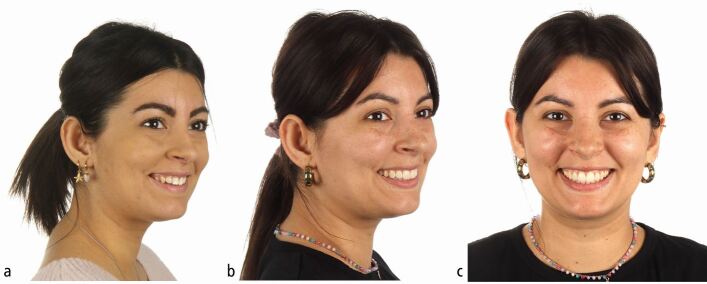
a, b, c) Smile projection, smile width and smile line were greatly improved by orthodontic-periodontal-restorative treatment

**Fig. 8:**
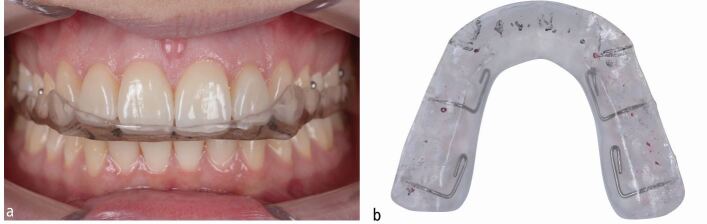
a, b) A Kois splint was delivered to the patient for nighttime use to protect the restorations and to prevent further tooth wear due to reported nocturnal bruxism^21^

By combining pre-restorative orthodontic levelling and aligning with surgical crown lengthening, the biological cost was minimised while achieving excellent dental aesthetics and function. The worn lower incisors were restored with composite material to establish interincisal contact during static and dynamic occlusion (Fig. 6, Fig. 7). Given the history of parafunctional activity, a custom-made, maxillary, Kois occlusal splint for nighttime wear was delivered to reduce the risk of restorative failure (Fig. 8).^21^

## Missing teeth

In contemporary practice, adult patients are often referred to the orthodontist for fast uprighting of tipped teeth adjacent to edentulous areas, coupled with the request for limited ‘invisible' clear aligner pre-prosthodontic therapy before ceramic veneers. These ‘limited' orthodontic pre-treatments which allow the prosthodontist to commence rehabilitation efficiently are limited in their scope to address substantial malocclusions. A comprehensive interdisciplinary approach directed at comprehensive management of all dental, skeletal and functional issues may be more time-consuming. The zeitgeist of limited-objective, clear aligner treatment, often provided by general practitioners, may have impacted the willingness to undergo comprehensive interdisciplinary therapy delivered by skilled interdisciplinary teams of specialists.

### Case example

**Fig. 9:**
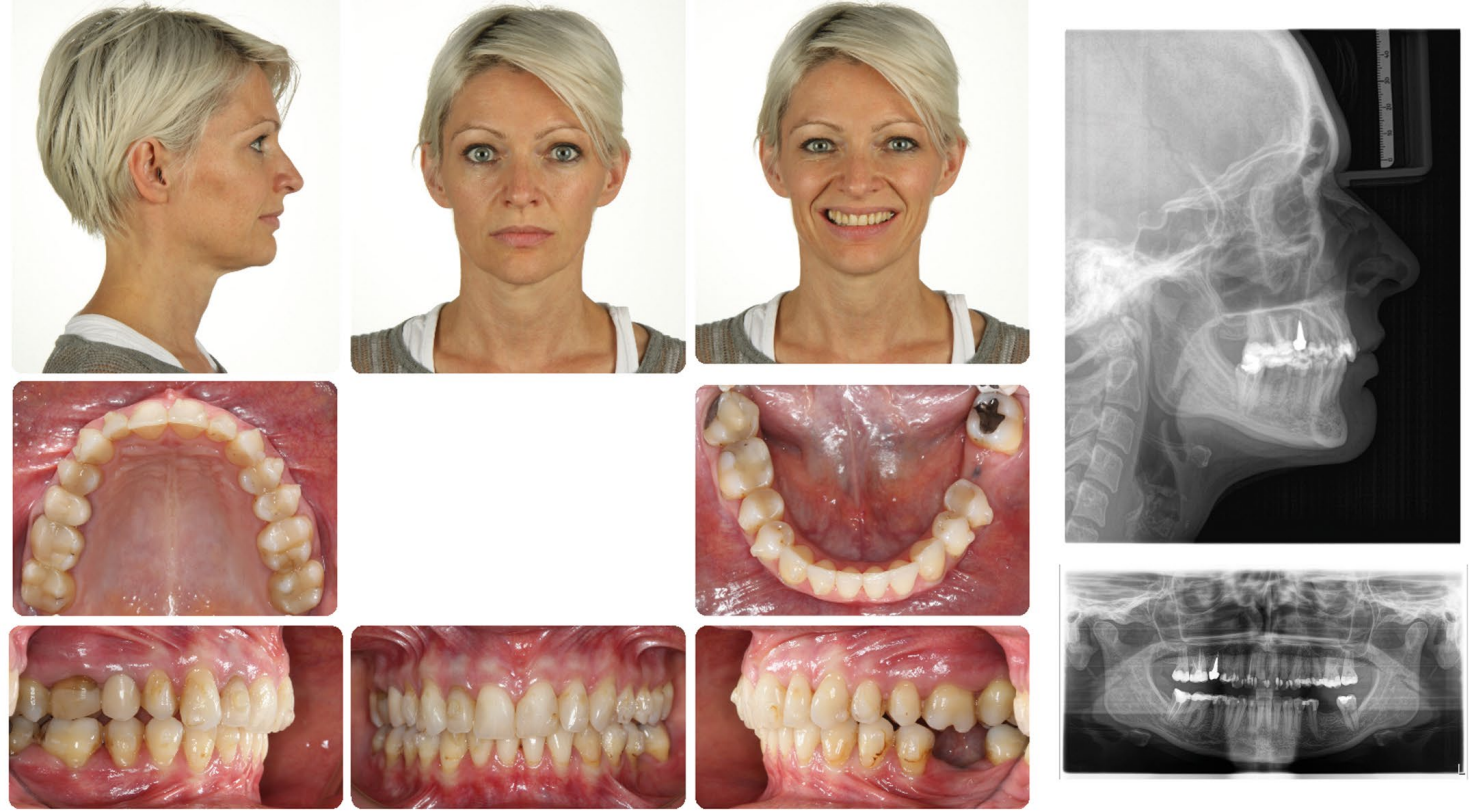
This 40-year-old patient had been in clear aligner treatment for two years *alio loco* and was not satisfied with the result. Due to her high aesthetic demand and the asymmetrical skeletal Class II malocclusion, a comprehensive orthodontic-surgical-perio-prosthodontic treatment plan was established, which included a bi-sagittal split osteotomy for mandibular advancement with midline correction

A 40-year-old female patient presented dissatisfied with the result of two-year clear aligner treatment and complained about residual anterior dental malalignment and the sensation of ‘an uncomfortable bite'. Clinical inspection revealed an asymmetric Class II Division 2 malocclusion with small upper lateral incisors, a 3 mm mandibular midline deviation to the left and a missing lower left first permanent molar. There were inadequate restorations and multiple gingival recessions. The panoramic radiograph showed several root canal treatments, moderate apical root resorptions predominantly of the maxillary incisors, and a smaller left mandibular condyle. The cephalometric analysis revealed a skeletal Class II pattern due to mandibular retrognathia and normal vertical skeletal proportions (Fig. 9).

**Fig. 10:**
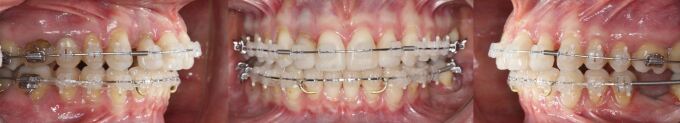
Orthodontic dental alignment, levelling and decompensation was necessary to achieve sufficient overjet for surgical mandibular advancement with centring of the lower midline

**Fig. 11:**
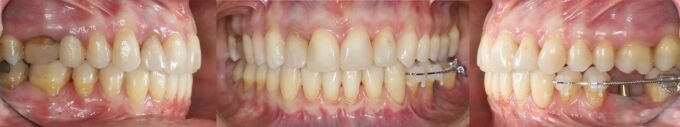
Orthodontic-surgical treatment has established a solid bilateral Class I occlusion and centred midlines

All existing clear aligner attachments were removed and the patient started fixed appliance treatment with levelling, aligning and dental decompensation to allow for subsequent surgical mandibular advancement with rotation to the right side to establish bilateral Class I occlusal relationships and correction of the lower midline (Fig. 10, Fig. 11).

**Fig. 12:**
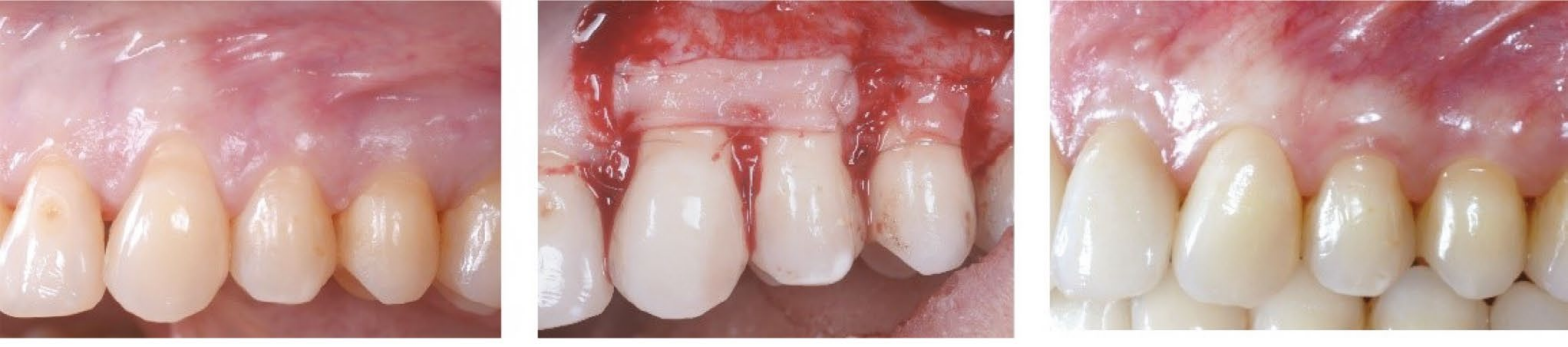
Several connective tissue grafts were necessary due to generalised gingival recessions

**Fig. 13:**
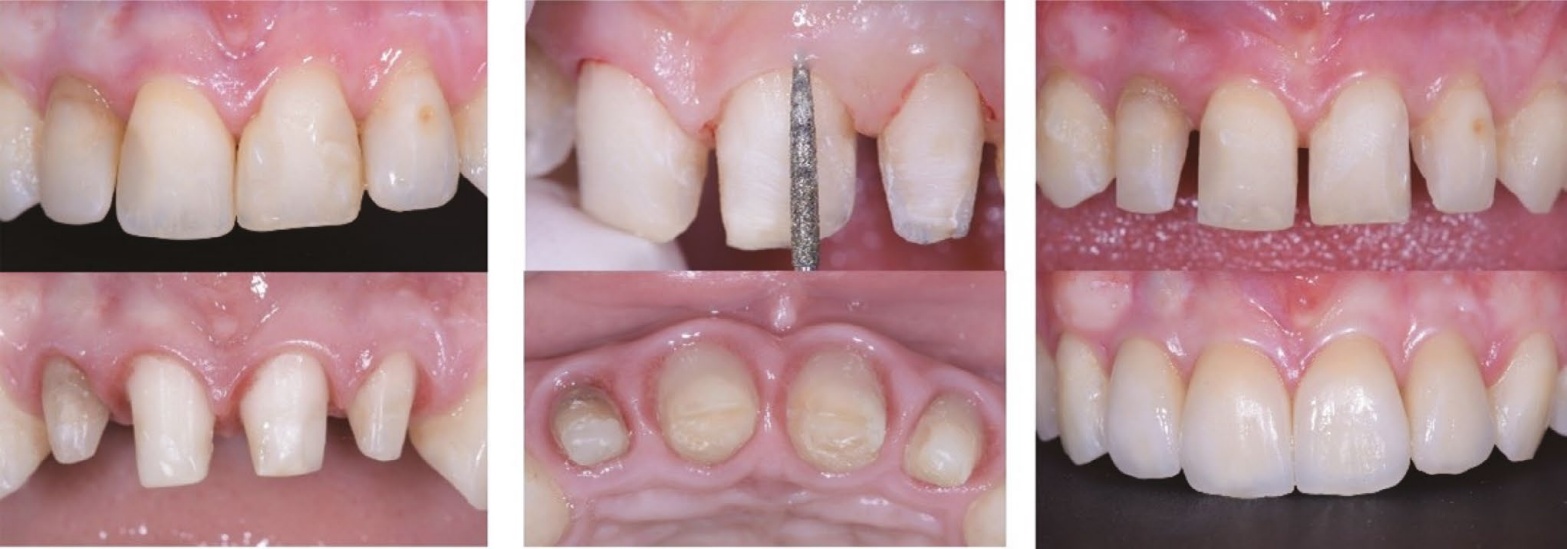
In the upper aesthetic zone, the extremely thin periodontal biotype required tooth preparation according to E. Loi (‘gingitage') to create slightly thicker and harmonious gingival conditions before delivery of four ceramic crowns^22^

**Fig. 14:**
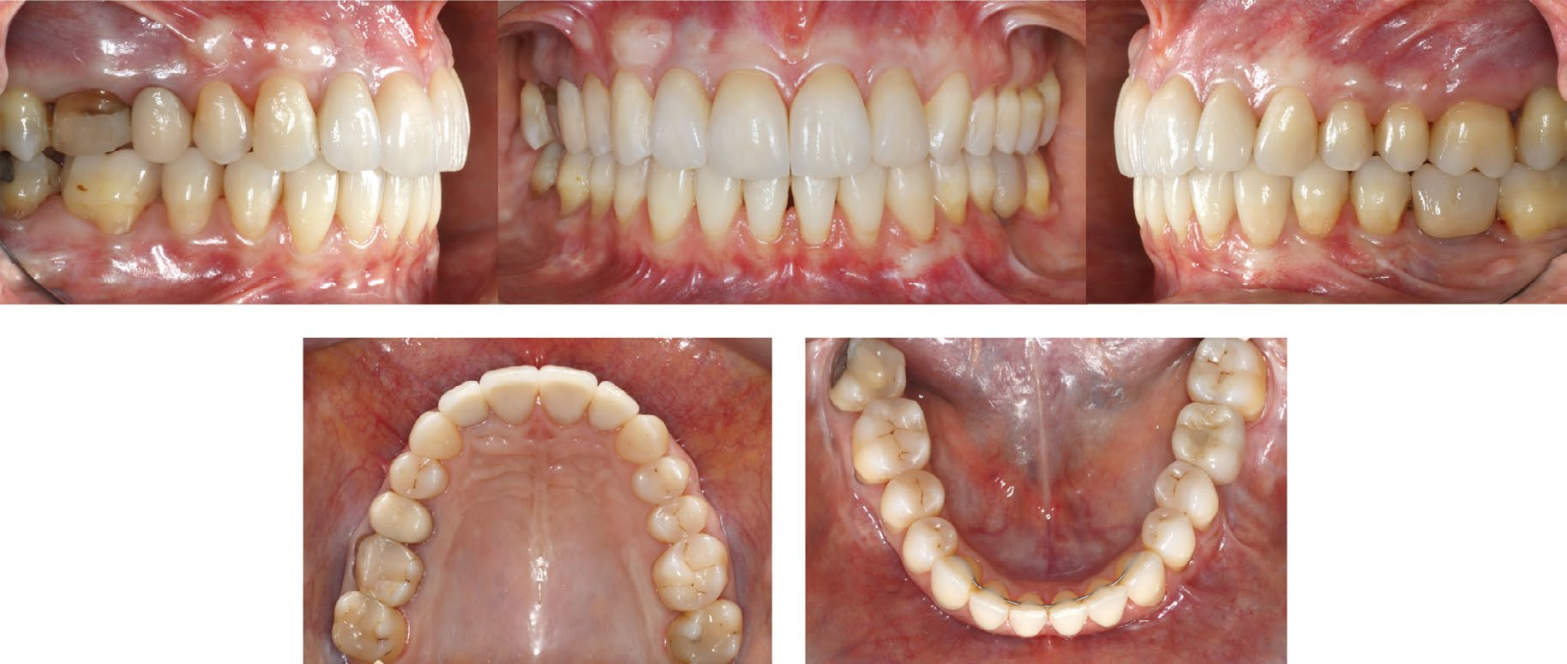
An excellent overall result has been achieved which has comprehensively addressed all dental, periodontal, skeletal and functional aspects. Due to economic limitations, a new crown for tooth 16 had to be postponed

After removal of the appliances and basic dental care, mucogingival surgery with connective tissue grafting was performed to address the gingival recessions (Fig. 12). Biologically oriented preparation of the maxillary incisors to enhance the gingival conditions before placement of four ceramic maxillary incisor crowns was performed to maximise the aesthetic and periodontal outcome in the sensitive anterior aesthetic zone (Fig. 13).^22^ An implant-borne crown for substitution of the missing lower left first molar was inserted, while a crown on the maxillary right first molar is planned (Fig. 14).

**Fig. 15:**
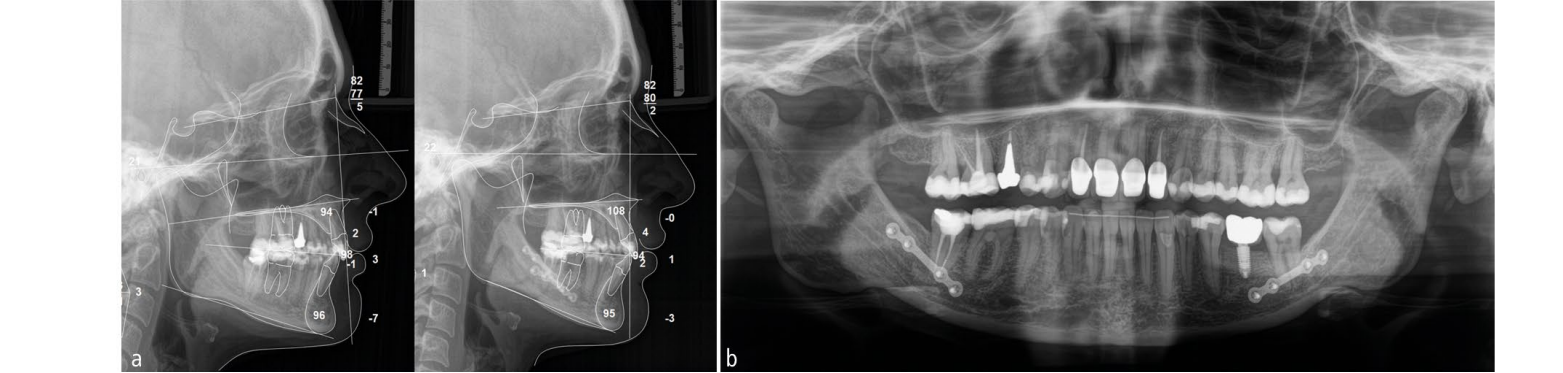
a, b) Pre- and post-treatment cephalometric analysis and orthopantomogram after comprehensive interdisciplinary care

**Fig. 16:**
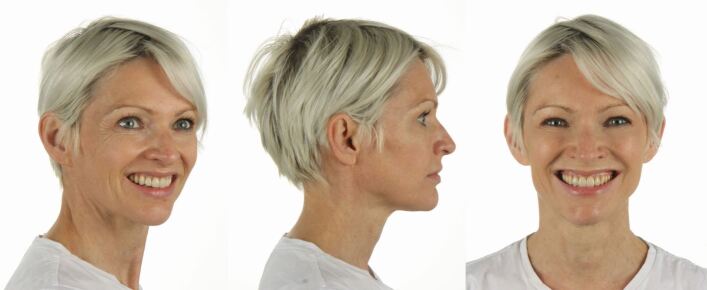
The patient is completely satisfied with her facial and dental aesthetics

The interdisciplinary orthodontic-surgical-periodontal-restorative-prosthodontic treatment has comprehensively addressed the dentoskeletal issues leading to complete patient satisfaction two years after the end of orthodontic-surgical treatment (Fig. 15, Fig. 16).

## Dental trauma with avulsion

Avulsion of permanent teeth has been reported in 0.5-3% of all dental injuries, with a higher prevalence in children and teenagers.^23^^,^^24^ In adults, the most frequent causes of traumatic dental injuries are accidental falls (40%), sports activities, cycling and traffic accidents.^25^ The maxillary central incisors are the most frequently traumatised teeth, followed by the maxillary lateral incisors. As reported, lateral luxation is the most prevalent luxation injury (40%) followed by avulsion (17-25%).^25^^,^^26^

If teeth in the aesthetic zone are lost due to trauma, the labial soft tissues and the alveolar plates may also be affected, contributing to a subsequent deficiency of the alveolar crest for implant placement. Satisfactory aesthetics may require repair of the damaged tissues with hard and soft tissue grafts. Orthodontic tooth movement into the edentulous area may assist in regaining both hard and especially soft tissue, reducing the need for extensive grafting and improving aesthetics. This approach should therefore be considered as part of interdisciplinary care.

### Case example

**Fig. 17:**
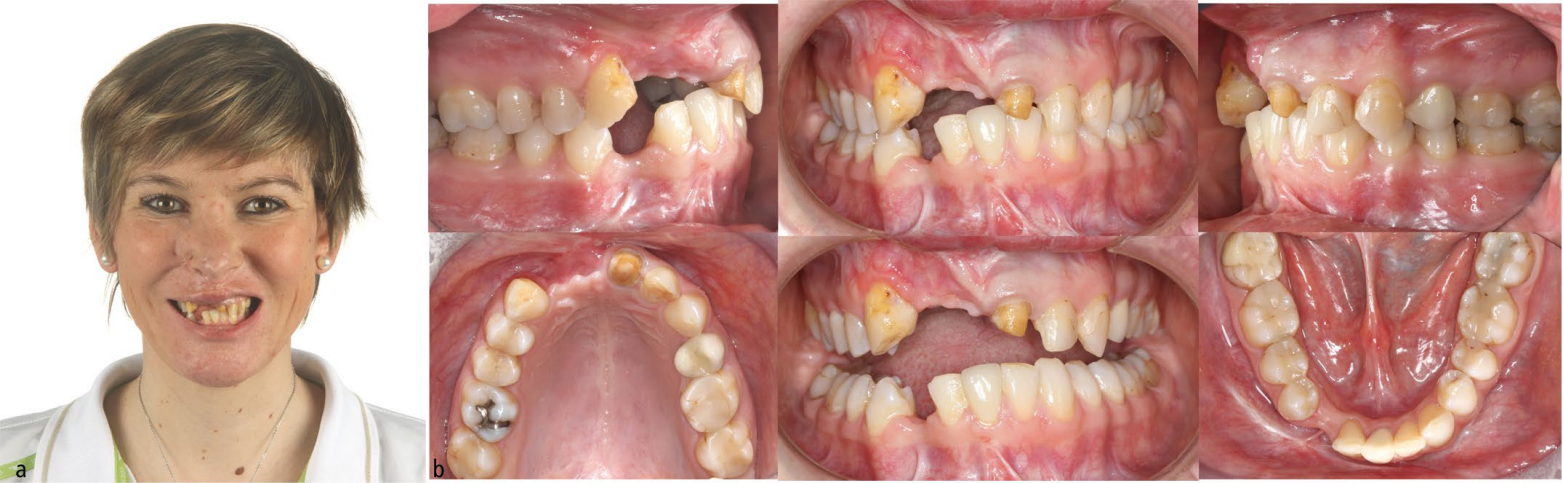
a, b) Multiple traumatic tooth loss of the right maxillary central and lateral incisors and the lower right canine, and a crown fracture of the left maxillary central incisor with a massive hard and soft tissue defect and scarring. Note that the upper left first premolar and the lower right lateral incisor had been previously extracted for orthodontic purposes *alio loco*

**Fig. 18:**
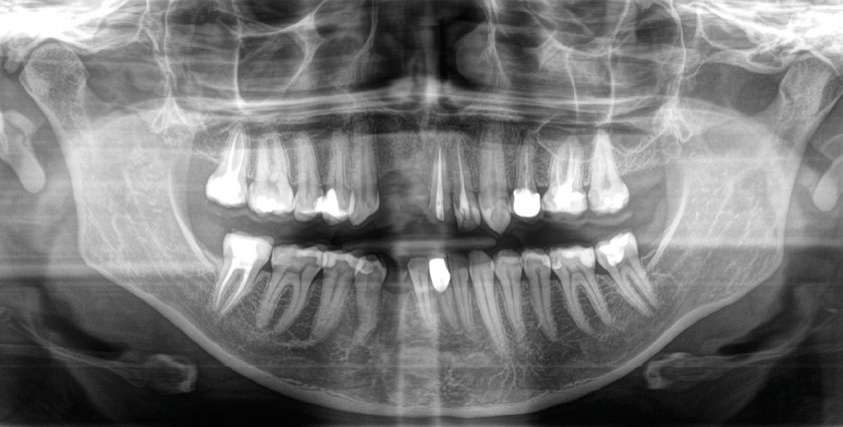
The panoramic radiograph reveals a massive osseous defect in the anterior maxilla

A 38-year-old woman was referred to the orthodontist after a bicycle accident involving avulsion of the maxillary right central and lateral incisor, and crown fractures of the upper right canine and the left central incisor. A significant hard and soft tissue defect and substantial scarring in the edentulous area were noted. The mandibular right canine was also lost during the trauma, with associated buccal bony resorption apparent. Significant scarring of the upper lip, the philtrum and the tip of the nose was also present. The patient had a history of previous orthodontics during adolescence involving the extraction of the upper left first premolar and the lower right lateral incisor. The canine relationships were Class I bilaterally with a Class I molar occlusion on the right and a full Class II molar relationship on the left side due to previous extraction of the maxillary right first premolar (Fig. 17, Fig. 18).

Following interdisciplinary treatment planning, a decision was made to avoid dental implants in the severely damaged aesthetic zone and to reduce the size of an anterior bridge to a single pontic by orthodontic mesialisation of the right canine into the lateral incisor area. This procedure would lead to the creation of hard and soft tissue in the edentulous area, improving emergence profile and gingival aesthetics. An implant-borne crown was planned to substitute the mesialised canine. In the lower arch, mesialisation of the right first premolar into the deficient site of the avulsed canine was planned to improve the bony support for an implant-borne premolar crown by orthodontic site development.

**Fig. 19:**
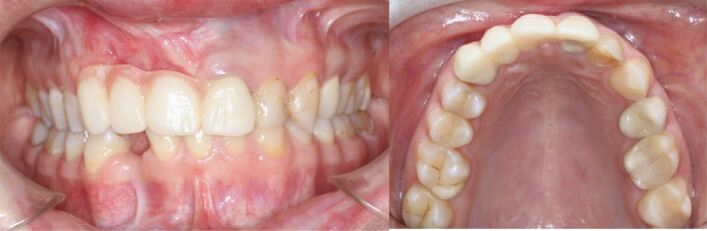
A provisional resin bridge was delivered for improving dental aesthetics

**Fig. 20:**
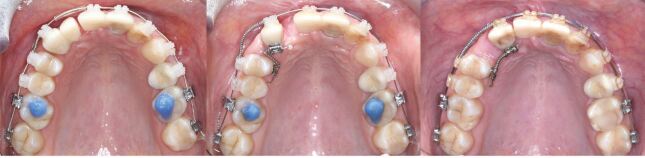
The upper right canine was mesialised with ‘tandem' mechanics into the lateral incisor area with the aim to create hard and soft tissue and to open space for a canine implant

**Fig. 21:**
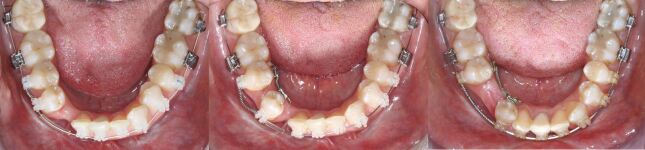
The lower right first premolar was mesialised into the lateral incisor area to improve the mesial osseous defect before implant placement

After conservative and endodontic treatment, a four-unit temporary resin bridge from 13 to 21 was placed (Fig. 19). Fixed appliance treatment with open coil springs and tandem mechanics on the lingual aspect was initiated to bodily mesialise the upper right canine and the lower right premolar. The temporary bridge was separated and the pontic was sequentially reduced. Rebracketing of the canine on two occasions was necessary to promote root parallelism (Fig. 20, Fig. 21).

**Fig. 22:**
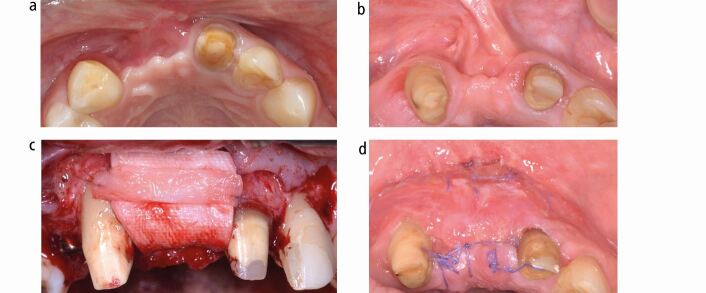
a, b, c, d) Despite having gained some soft tissue by canine mesialisation, both hard and soft tissue grafting of the anterior defect were necessary before delivery of the final bridgework

**Fig. 23:**
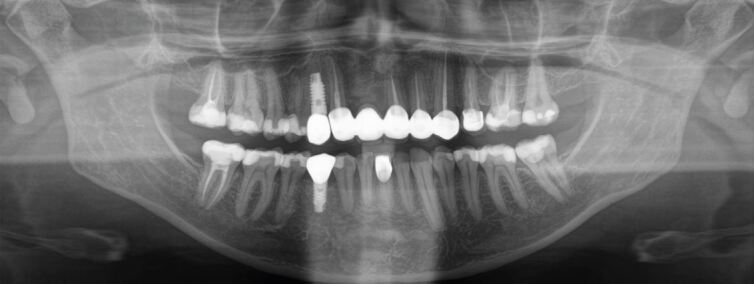
Orthopantomogram after the end of orthodontic-perio-prosthodontic therapy with stable osseous conditions after insertion of two implant-borne crowns and an anterior five-unit ceramic bridge

**Fig. 24:**
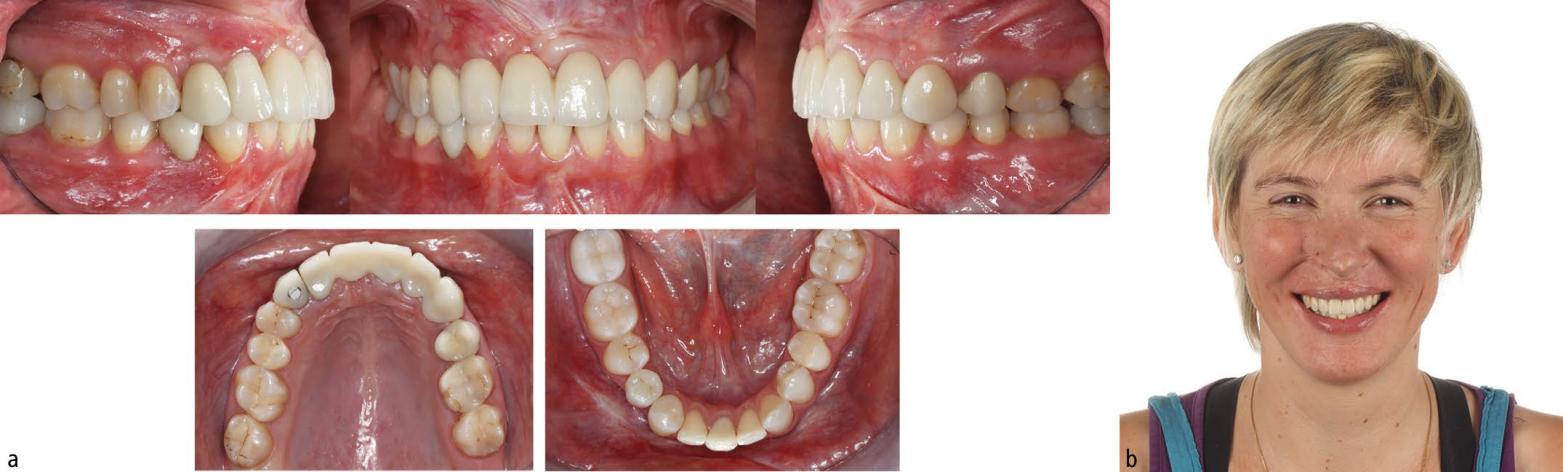
a, b) Solid Class I molar and canine relationships with three lower incisors and the mesialised lower right first premolar replacing the lost canine. The severe traumatic hard and soft tissue defect has been recuperated and despite the nose and upper lip scarring smile aesthetics are very satisfactory

An improvement of the soft tissue contour around the upper mesialised canine arose and sufficient bony support for insertion of an implant was created. Hard and soft tissue grafting of the incisor area is still necessary before delivery of the anterior bridge to enhance both the vertical and horizontal dimension of the alveolar crest, albeit to a lesser extent. In the lower arch, no additional grafting was required before implant placement after mesialisation of the first premolar. Reducing the span of the upper anterior bridge avoiding two adjacent implants in the sensitive aesthetic zone facilitated a less invasive and more predictable rehabilitation, while also satisfying aesthetic expectations despite the severely mutilated hard and soft tissues (Fig. 22, Fig. 23, Fig. 24).

## Functional issues coupled with high dentofacial aesthetic demands

Obstructive sleep apnoea (OSA) is a highly prevalent and under-diagnosed condition, affecting at least 4% of men and 2% of women.^27^ The disorder is characterised by recurrent episodes of upper airway obstruction and is associated with reduction in ventilation, resulting in recurrent arousals and episodic oxyhemoglobin desaturations during sleep.^28^ Structural changes, including tonsillar hypertrophy, retrognathia and variations in craniofacial structures, have been linked to an increased risk of sleep apnoea, likely by increasing upper airway collapsibility. Maxillomandibular advancement surgery (MMAS) in combination with concomitant maxillary expansion may be considered for adults with more severe forms of OSA to improve the function and quality of life. A positive effect on the quality of life and success rates for MMAS of 57-100% due to increased upper airway volume have been reported.^29^^,^^30^^,^^31^^,^^32^^,^^33^^,^^34^

### Case example

**Fig. 25:**
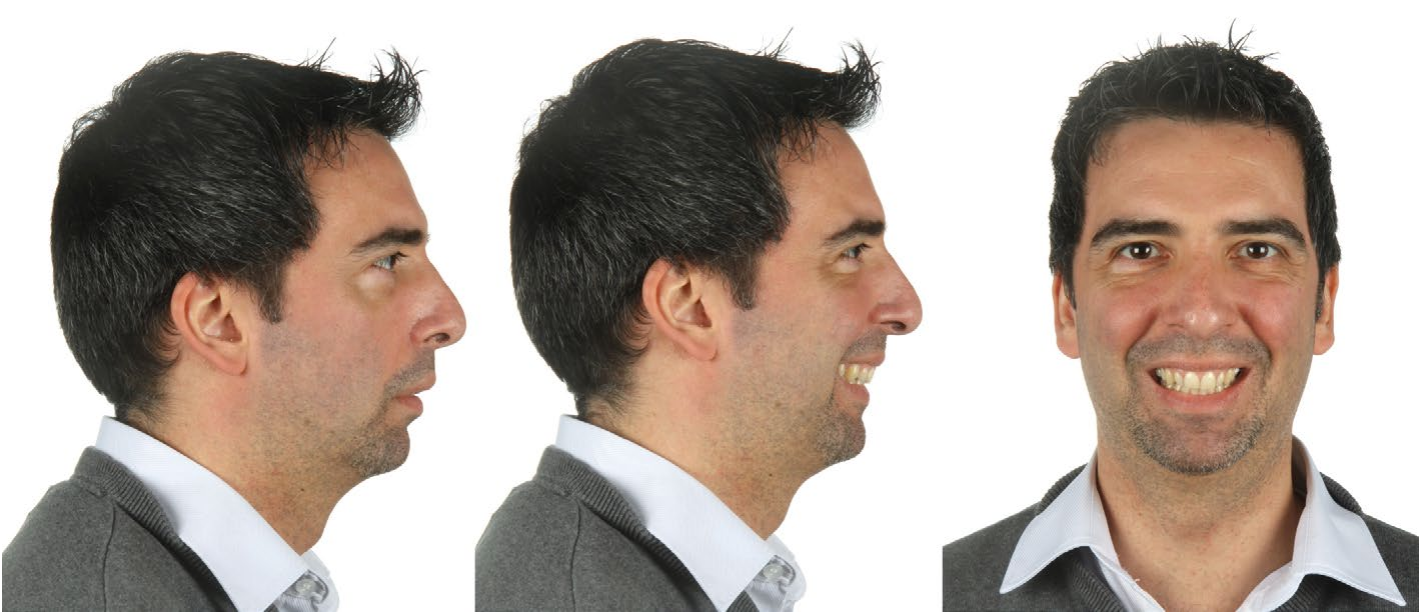
This 38-year-old male patient was affected by mild OSA with severe mandibular retrusion, a constricted smile and retroclined maxillary incisors, and requested both functional and major aesthetic improvement

**Fig. 26:**
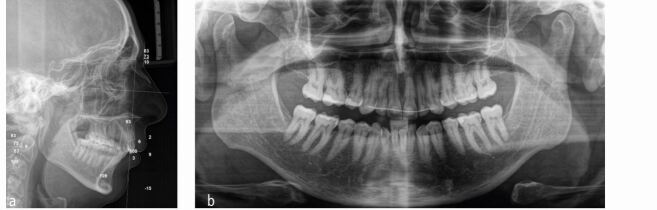
a, b) The radiographs show a marked high-angle skeletal Class II malocclusion with an ANB angle of 10 and dentoalveolar compensation after extraction of the upper first premolars

A 38-year-old male patient was referred by an ear, nose and throat surgeon with a diagnosis of moderate OSA characterised by an Apnoea Hypopnea Index score of 15 based on polysomnography. The patient complained about daytime sleepiness and was unsatisfied with facial and smile aesthetics, especially in relation to deficient chin projection and transverse maxillary constriction. Significant mandibular retrognathia and an increased lower anterior facial height was present (Fig. 25, Fig. 26). He reported being unable to tolerate mandibular advancement splint therapy.

**Fig. 27:**
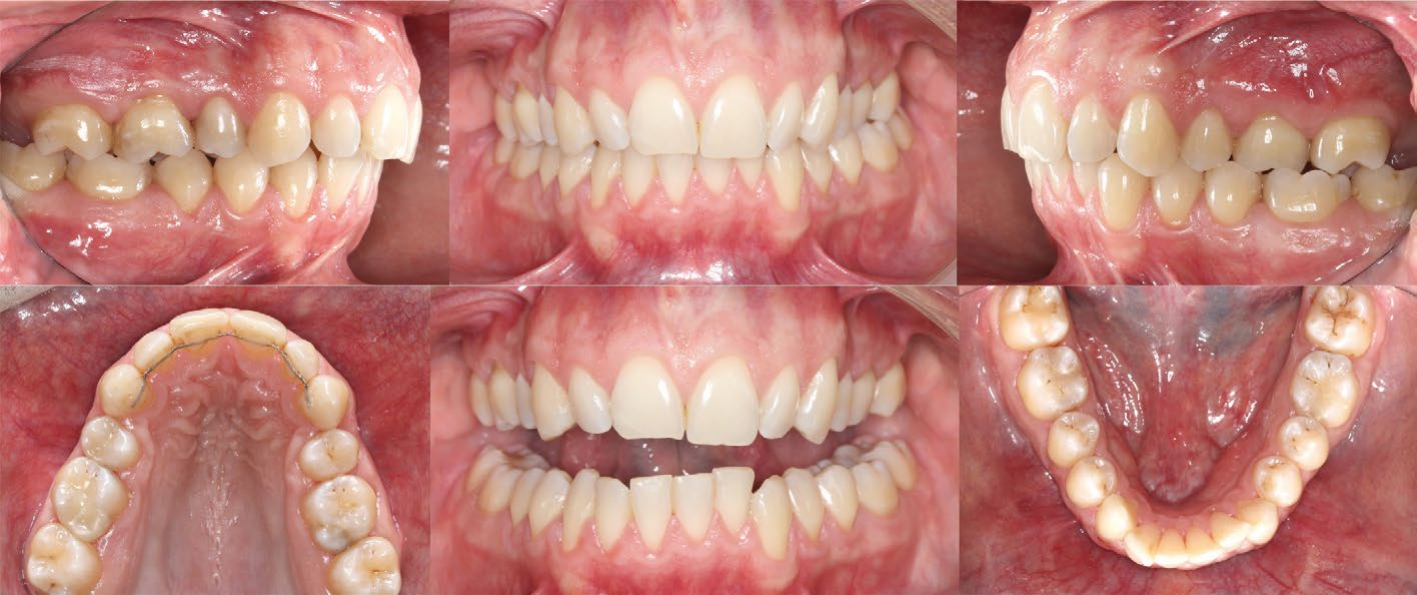
Due to former orthodontic two-premolar extraction camouflage treatment, the molars are in a full Class II relationship. Both arches are constricted with an accentuated lower curve of Spee and mandibular crowding. Note dental wear of the upper and lower anterior teeth

The canines were in a Class I relationship, with the molar relationships being Class II secondary to a primary course of orthodontic camouflage involving extraction of the upper first premolars during early adulthood. The maxillary incisors were retroclined with incisal wear and maxillary arch constriction, with a resultant crossbite involving the left first molar. The upper lateral incisors and the second premolars were diminutive contributing to a Bolton discrepancy. An accentuated lower curve of Spee was present with anterior crowding (Fig. 27).

**Fig. 28:**
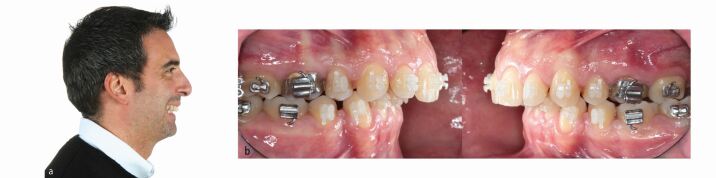
a, b) The lower first premolars were extracted for pre-surgical dentoalveolar decompensation to allow for sufficient mandibular advancement

In view of the functional and facial concerns, a combined orthodontic-surgical-restorative treatment was recommended. As the amount of maxillary incisor proclination for pre-surgical dental decompensation alone would not produce sufficient overjet to permit meaningful surgical mandibular advancement, extractions of the lower first premolars were undertaken to level, align and retract the mandibular incisors (Fig. 28). The alternative strategy to re-open upper premolar spaces for two implant-borne crowns was rejected by the patient. However, restoration of both the small lateral incisors and second premolars with direct composite would be required to avoid residual interdental spaces and enhance the dental aesthetics.

**Fig. 29:**
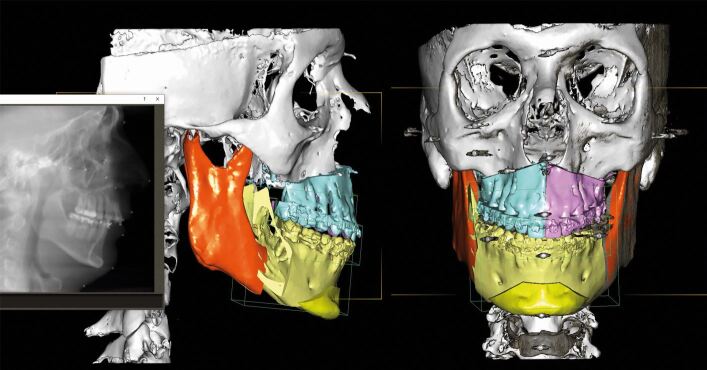
Virtual 3D planning of bimaxillary advancement with maxillary expansion and genioplasty

**Fig. 30:**
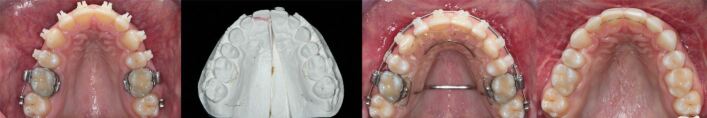
Surgical posterior expansion of 6 mm by a two-piece Le Fort I osteotomy was necessary to avoid a post-surgical posterior crossbite

**Fig. 31:**
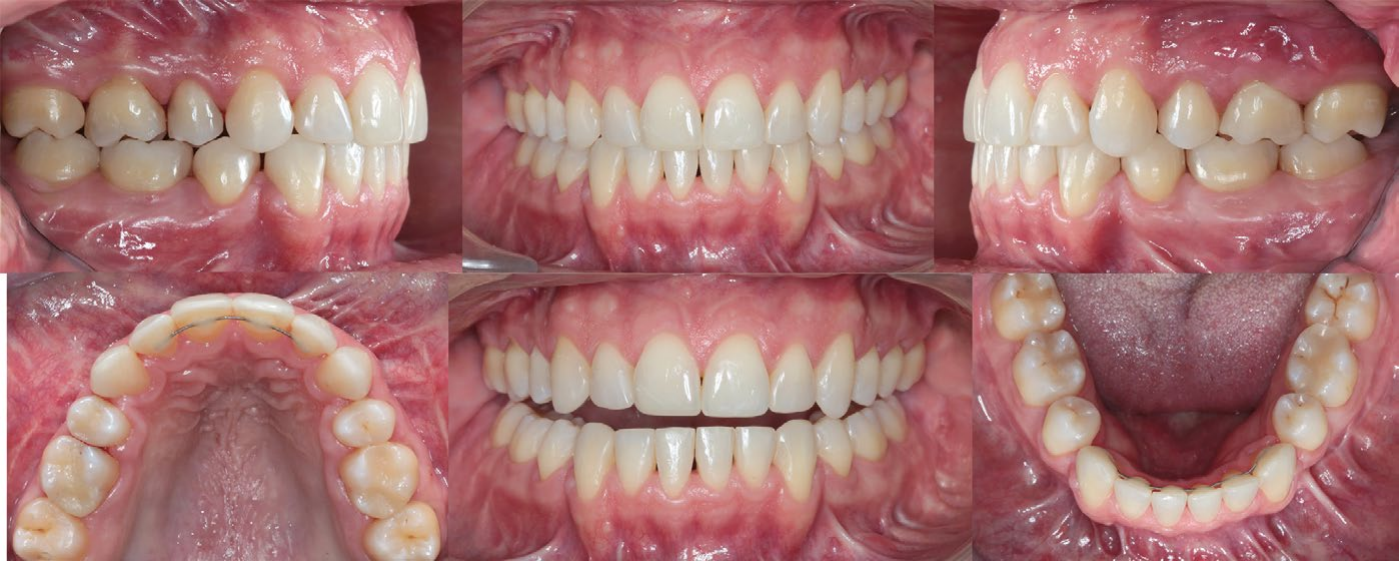
A solid Class I occlusion with complete levelling of the curves of Spee has been achieved and the small upper lateral incisors, the second premolars and the worn incisal edges and canine cusps have been restored with composite material for optimising both aesthetics and function

**Fig. 32:**
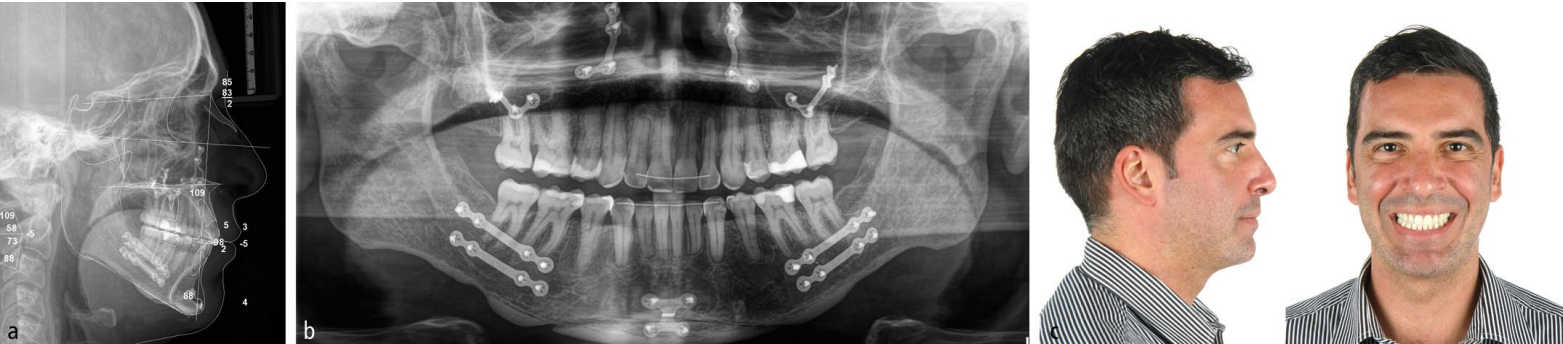
a, b, c) Bimaxillary surgical advancement with a genioplasty and maxillary expansion has corrected the severe skeletal Class II malocclusion and has led to a pleasing profile with significant improvement of smile aesthetics

**Fig. 33:**
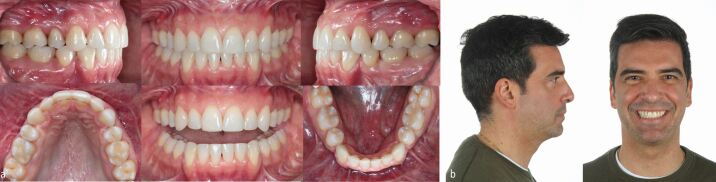
a, b) Good overall maintainability of the overall result three years after the end of treatment. The patient is very satisfied with the aesthetic and functional improvement

After 15 months of presurgical orthodontics, virtual 3D digital planning of a two-piece Le Fort I osteotomy, involving 6 mm of maxillary posterior expansion and 2 mm advancement and a bilateral sagittal split osteotomy with a genioplasty introducing a 10 mm mandibular advancement, was performed (Fig. 29, Fig. 30). Following four months of post-surgical stabilisation and orthodontic finishing, the appliances were removed and composite restorations on the distal aspect of the diminutive maxillary lateral incisors and on the mesial aspect of the second premolars were performed. All worn incisal edges and canine cusps were also restored (Fig. 31). A pleasing facial and occlusal outcome was obtained, with the skeletal Class II deformity and vertical excess being corrected (Fig. 32). Post-treatment polysomnography also revealed a reduction of the Apnoea Hypopnea Index score from 15 to 4. There was also associated subjective improvement in daytime sleepiness with a commensurate improvement in quality of life. Three years following the completion of interdisciplinary treatment, excellent levels of stability were observed (Fig. 33).

## Conclusion

Adult orthodontics has become increasingly ingrained and will continue to gain momentum worldwide due to the ageing population and increased access to treatment. The baby boomers' intrinsic demand to look good at an advanced age, together with greater social pressure, more public awareness of the existing possibilities to improve both aesthetic and functional impairment with ‘invisible' orthodontics, and the advent of minimally invasive restorative treatment and less invasive surgery, has opened new horizons for highly skilled, interdisciplinary teams. Complex adult treatment is benefited by close collaboration between well-trained dental specialists to diagnose, plan and execute the most suitable treatment for the individual patient. Combining solid basic orthodontic knowledge with the latest digital technology and continuous immersion into the complementary dental and medical disciplines will ensure a bright professional future for the next generation of orthodontic specialists and ensure that adult patients with multi-faceted demands are treated to the highest possible standard.
